# Morphological and Structural Details of Tomato Seed Coat Formation: A Different Functional Role of the Inner and Outer Epidermises in Unitegmic Ovule

**DOI:** 10.3390/plants11091101

**Published:** 2022-04-19

**Authors:** Inna A. Chaban, Alexander A. Gulevich, Neonila V. Kononenko, Marat R. Khaliluev, Ekaterina N. Baranova

**Affiliations:** 1 Plant Cell Biology Laboratory, All-Russia Research Institute of Agricultural Biotechnology, Timiryazevskaya 42, 127550 Moscow, Russia; nilava@mail.ru; 2 Laboratory of Plant Cell Engineering, All-Russia Research Institute of Agricultural Biotechnology, Timiryazevskaya 42, 127550 Moscow, Russia; a_gulevich@mail.ru (A.A.G.); marat131084@rambler.ru (M.R.K.); 3 Department of Biotechnology, Institute of Agrobiotechnology, Russian State Agrarian University—Moscow Timiryazev Agricultural Academy, Timiryazevskaya 49, 127550 Moscow, Russia; 4 N.V. Tsitsin Main Botanical Garden of Russian Academy of Sciences, Botanicheskaya 4, 127276 Moscow, Russia

**Keywords:** seed coat, integument, epidermis, protective functions, cell modifications, differences in development, ricinosomes, cell wall lignification

## Abstract

In order to understand how and what structures of the tomato ovule with a single integument form the seed coat of a mature seed, a detailed study of the main development stages of the tomato ovule integument was carried out using the methods of light and electron microscopy. The integument itself it was shown to transform in the course of development into the coat (skin) of a mature seed, but the outer and inner epidermises of the integument and some layers of the integument parenchyma are mainly involved in this process. The outer epidermis cells are highly modified in later stages; their walls are thickened and lignified, creating a unique relatively hard outer coat. The fate of the inner epidermis of integument is completely different. It is separated from the other parenchyma cells of integument and is transformed into an independent new secretory tissue, an endothelium, which fences off the forming embryo and endosperm from the death zone. Due to the secretory activity of the endothelium, the dying inner parenchyma cells of the integument are lysed. Soon after the cuticle covers the endosperm, the lysis of dead integument cells stops and their flattened remnants form dense layers, which then enter the final composition of the coat of mature tomato seed. The endothelium itself returns to the location of the integument inner epidermis.

## 1. Introduction

Studying the ontogeny of almost any plant seed is a unique opportunity for the simultaneous research of two opposite processes: cell division and differentiation on the one hand, and the death of the same cells on the other hand [1]. In addition, such an investigation makes it possible to trace the process of coat (skin) formation in a mature seed and the degree of participation in this process of specific cell layers or tissues.

Tomato (*Solanum lycopersicum* L.) is one of the most important food crops, ranking first in the world among vegetable products in terms of its importance and volume of production [2]. The fruit of tomato is a fleshy multi-nested berry. Many ovules form in each nest on the placenta. The tomato ovule is anatropic, with single multilayered integument. Thus, the tomato ovule belongs to the unitegmic ovules. The formation of a full-fledged seed coat of any plant is an important condition for the effective and safe development of the embryo, and for the preservation of mature seeds [3]. The integrity and protective properties of the seed coat are extremely important for the quality and suitability of the seed during storage, and to prevent damage from various processing technologies. Hence, knowledge of the features and details of the formation of the seed coat (skin) can be extremely useful for monitoring the implementation of the genetic potential of new hybrids and varieties. The traditional understanding of the role of the seed coat is that it provides protection for the developing embryo and endosperm [4].

A number of reviews analyzing studies of the structure and functional role of the seed coat (skin) in various plants [3,5,6] consider tissue transformation and cell death as a model for programmed cell death (PCD). It is assumed that the seed skin is a number of unique tissues that undergo differentiation during the seed formation, for the subsequent implementation of certain functions [5]. The seed coat is believed to physically restrict seed growth in a process that includes active responses to mechanical or biochemical signals sent by the developing endosperm [7]. Biochemical studies of a coat of different seeds have shown that a wide range of new compounds are synthesized in the seed coat, which are necessary for the successful ensuring of dormancy and germination [5]. Radchuk and Borisjuk (2014) evidenced that the function of the seed coat is to protect the embryo and at the same time transmit information about the external environment to the developing daughter tissues [3], wherein, the architecture, chemical composition, and metabolism of the seed coat work together to ensure that the ovule responds effectively to both biotic and abiotic factors. As a rule, the cell walls of the developing seed coat are rather strongly modified both due to thickening and due to the deposition of such polymers that reduce the permeability of the seed coat as lignin and suberin [8,9], in addition to proanthocyanidin [10] and other substances.

In our previous research, a detailed electron microscopic study of the ovule histogenesis in normal and genetically transformed tomatoes was carried out to reveal the cytoembryological causes of the endothelium growth and its transformation into pseudo-embryonic tissue in the ovules of transgenic tomatoes [11]. It was revealed that the mechanism of the detachment process of the inner epidermis from integument is the programmed death of adjacent parenchyma cells of the integument itself [11]. Ricinosomes, which are the markers of PCD, were found in these cells [12].

Nucellus cells in tomato ovule are destroyed by the time of fertilization. As a result, before fertilization, the embryo sac is directly adjacent to the inner epidermis of integument. The outer epidermis of integument also plays an important role in the formation of the coat of a mature tomato seed. Both of these transformations are probably equally important for the formation of the solid coat of mature seed [13,14].

PCD is a fundamental process in ensuring plant development. Despite the significant advances in recent investigations, it is still difficult to assess all aspects of the PCD role in the formation of individual tissues, modes of regulation, and signal transmission mechanisms [15,16]. A particular problem is the study of this process in developing vegetative reproductive organs, where simplified notions of the simultaneous death of specialized cells were the traditional concept [17,18]. These facts were considered ignoring the balance between proliferation and differentiation [19]. At present, it is already obvious that the death of a separate tissue, a layer of cells, or an individual cell is a finely regulated process that will require an enumeration of many approaches both for description and for understanding of PCD regulation [20,21].

The main goal of this study was to research the morphological and ultrastructural details and the dynamics of formation of a sufficiently solid tomato seed coat (skin) and to identify which ovule tissues are involved in this process. Since the ovule development is inevitably associated with the death of integument parenchyma cells, the thorough description of this process at the ultrastructure level is part of this work.

## 2. Results

The tomato ovule has a single integument. The nucellus of the tomato ovule completely disappears by the fertilization period, and as a result the integument contacts directly with the embryo sac. By this time, the integument consists of 8–10 layers of parenchyma cells, enclosed within two protective layers—the outer and inner epidermis. After fertilization, the embryo and endosperm begin to form in the embryo sac. Free space for the embryo sac growth is formed due to expansion by programmed death of the inner layers of integument cells. However, the inner epidermis of the integument does not die. The inner epidermis is detached from the integument. This occurs through PCD of the integument parenchyma cells adjacent to the epidermis. In other words, the integument itself separates and alienates its own inner epidermis. As a result, its role and fate radically change: the inner epidermis of the integument is transformed into a new independent monolayer tissue—the endothelium.

The cells of the outer epidermis divide in a tangential direction, and the growth of the entire integument in the radial direction occurs mainly due to divisions of subepidermal cells. The endothelium, like a shield, separates and closes the developing new sporophyte from the zone of death and lysis of the parenchyma cells of the integument. Some details of this process are described in our previous work [11].

In the present study, we paid most attention to the anatomical and structural analysis of the formation and role of two opposite epidermises and the details of the PCD of the integument parenchyma cells of the tomato ovule in different stages of its development.

### 2.1. Dynamics Reorganization in the Integument Tissue of the Developing Seed (Light Microscopy)

**Figure 1:**
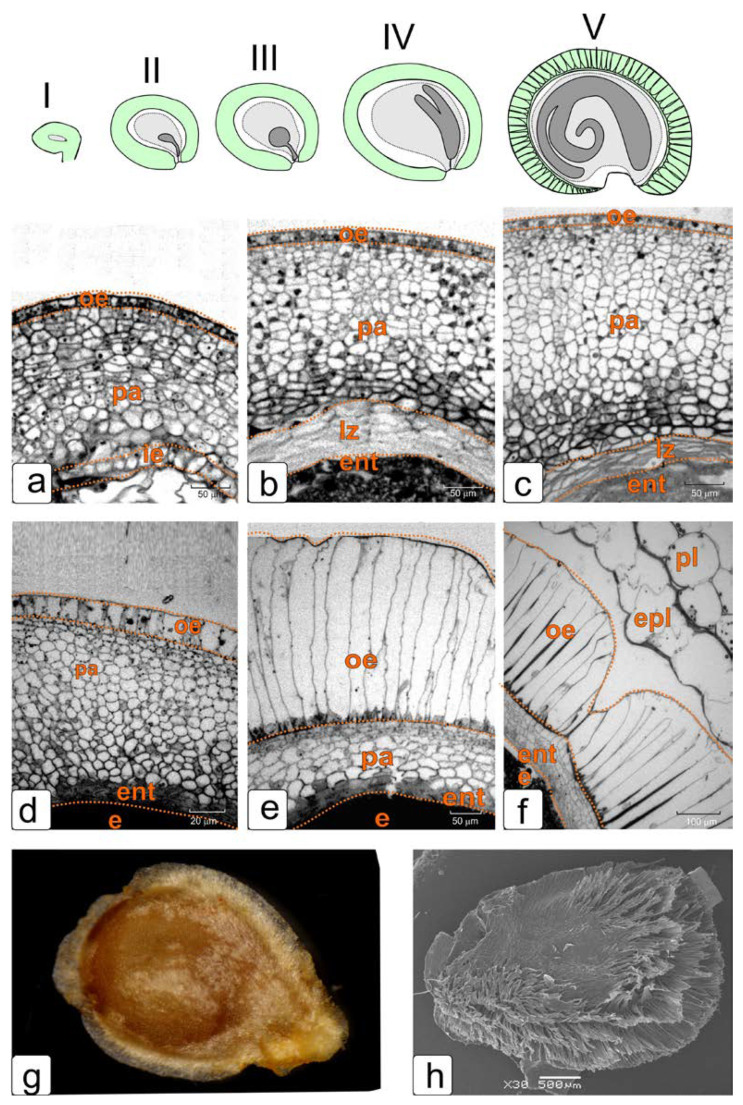
Light microscopy of fragments of integument longitudinal sections from the dorsal side of the tomato ovule in some key stages of ontogenesis. These stages are presented in the scheme (**I**–**V**). (**a**) Stage of a two-cell pro-embryo; (**b**) the stage of an early (pear-shaped) embryo; (**c**) the stage of the globular embryo; (**d**) stage of an early torpedo-shaped embryo; (**e**,**f**) the stage of a mature embryo; (**g**,**h**) the seed surface under a magnifying glass (**g**) and SEM (**h**). Abbreviations: e—endosperm, ie—inner epidermis, oe—outer epidermis, ent—endothelium, epl—placenta epidermis, lz—lysis zone, pa—integument parenchyma, pl—placenta.

The key stages of ontogenesis of the tomato ovule were mainly investigated in our study. They are shown in Figure 1 (schematic image of some key stages of ontogenesis I–V, top row) together with fragments of longitudinal sections of the integument from the dorsal side of the tomato ovule obtained by light microscopy (Figure 1a–f).

In the stage of early pro-embryo (and the beginning of endothelium formation) (Figure 1, top row, I), the integument contains 14–16 layers of parenchymal cells. (Figure 1a).

By the stage of an early embryo (Figure 1, top row, II), the number of parenchyma cell layers increases to 18–20. Simultaneously with this process, the zone of dying parenchymal cells expands on the inner side of integument (Figure 1b).

In the stage of globular embryo (Figure 1, top row, III), the integument of the tomato ovule contains the maximum number of layers of parenchymal cells—22–27 layers (Figure 1c, top row, III).

The cells of the outer zone of the integument are rather small. As the location of these cells approaches the embryo sac their size increases slightly. Most of the cells of the integument parenchyma are highly vacuolated.

In the next stages of development, the process of division of peripheral cells of the integument gradually stops, and the zone of lysis of dying cells continues to increase. The thickness of the entire integument layer begins to decrease accordingly. In the stage of a torpedo-shaped embryo, it is no more than 16 layers (Figure 1d,e). In this stage of ontogenesis, the changes begin to occur in the structure of the outer epidermis cells, associated mainly with a change in their shape and an increase in size (Figure 1d,e). Further, their growth occurs perpendicular to the outer surface. By the period of the embryo maturation, these cells are strongly elongated, and their envelopes begin to thicken. The integument parenchyma is reduced to 4–5 layers (Figure 1f).

**Figure 2:**
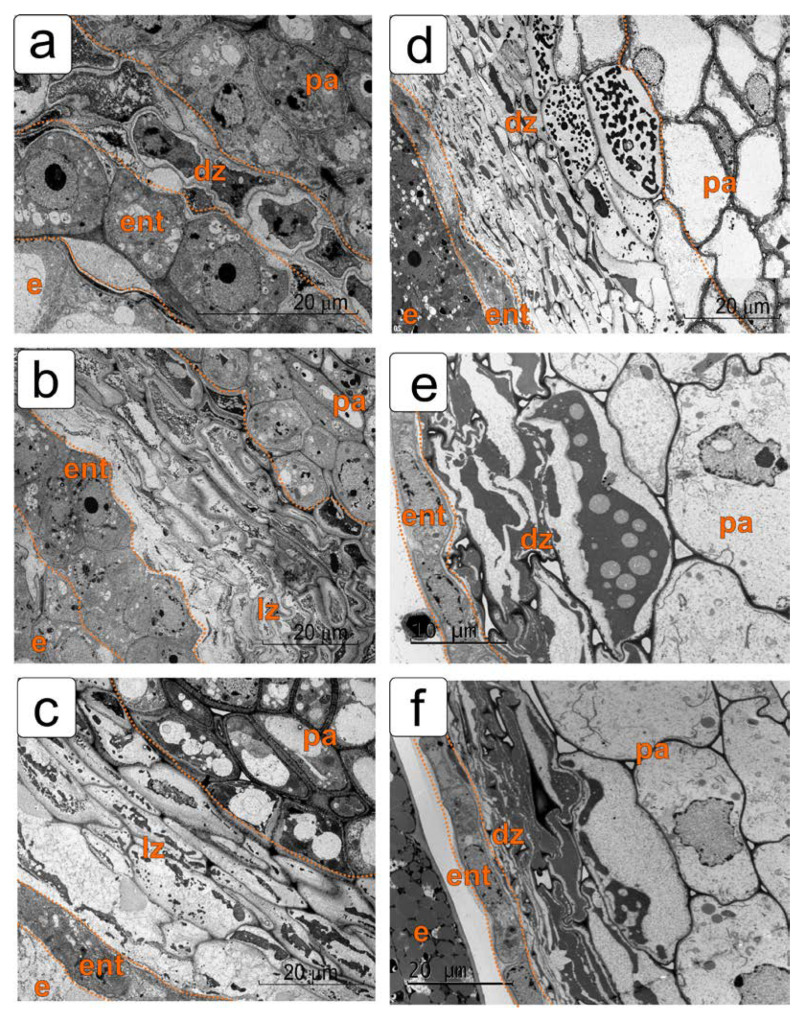
Transmission electron microscopy of ovule fragments of longitudinal sections in the endothelium and cell destruction of the integument in different development stages corresponding to images in Figure 1. (**a**) Stage of two-cell pro-embryo; (**b**) the stage of an early (pear-shaped) embryo; (**c**) stage of a globular embryo; (**d**) stage of a torpedo-shaped embryo; (**e**,**f**) stage of a mature embryo. Abbreviations: dz—cell death zone, e—endosperm, ent—endothelium, lz—lysis zone, pa—parenchyma.

Figure 2 shows fragments of the same sections of the tomato ovule in the PCD zone of integument and the functioning of the endothelium at the ultrastructure level.

In the early pro-embryo stage, the inner epidermis is completely separated from the rest of the integument cells by a layer of degraded cells, but still tightly adjoins the embryo sac (Figure 2a).

In the first stages of further development of the ovule, dying cells of the integument parenchyma move the formed endothelium away from the main part of the integument. However, for some time it remains in contact with the developing endosperm. In this stage, it is the endothelium that performs a protective function, protecting them from the harmful effects of the increasing lysis zone in relation to the integument and the developing embryo (Figure 2b).

By the globular embryo stage, a fairly wide zone of death and lysis of cells in the integument parenchyma is formed (Figure 2c). In this stage, the endothelium is also distanced from the endosperm. Further differentiation of the endothelium occurs correlated with the formation of endosperm, but independently of it. This is due to the formation of a lysis zone between both tissues. This process is described in detail in our previous work [11]. As PCD develops, most of the dying integument cells undergo lysis and disappear.

By the stage of the torpedo-shaped embryo, a cuticle is formed on the surface of the noticeably overgrown endosperm (Figure 2d). The fate of degrading protoplasts in dying integument cells changes. The protoplasts, compressed inside the envelopes, are then split into small bodies—vesicles, and already in the next stage they are again combined into a common mass (Figure 2d).

By the period of seed maturation, the remnants of cell walls and the contents of dead cells are compressed and strongly compacted. This layer borders directly on living endothelial cells (Figure 2e,f). The surface of the endosperm in this developmental stage is covered with a thick cuticular layer, which interrupts the contact between the new sporophyte and the inner epidermis of the endothelium. Further, the endothelium adjoins the compacted cells of the integument and develops independently of the endosperm (Figure 2e,f).

### 2.2. Features of the Endothelial Cells Structure in Different Stages of Seed Coat Formation

**Figure 3:**
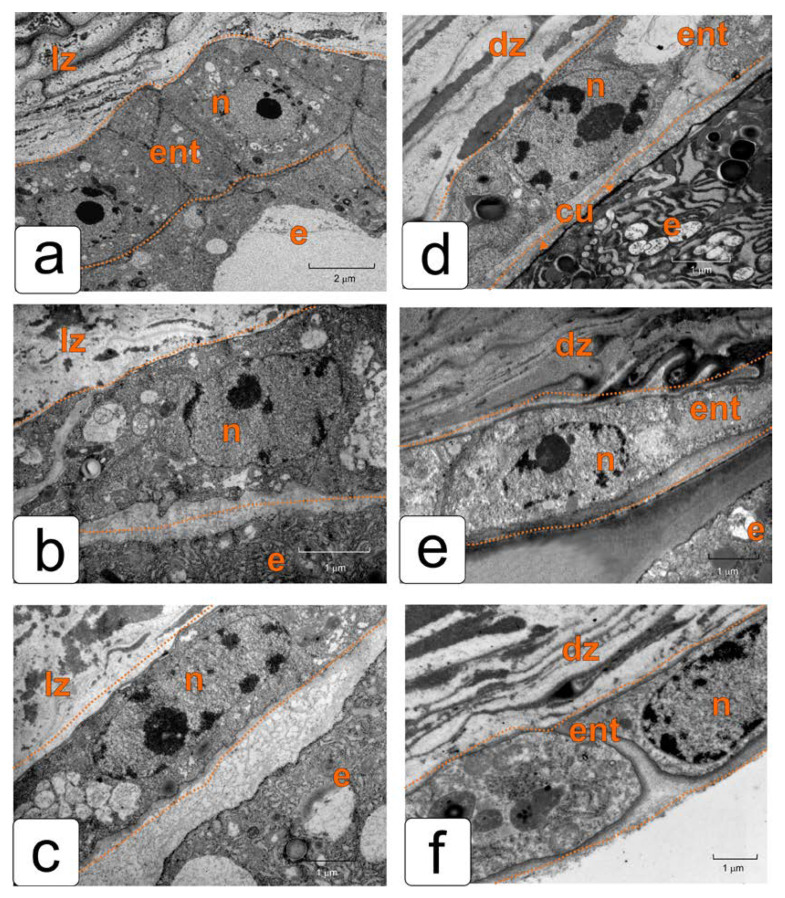
Ultrastructure changes in endothelial cells during the development of tomato ovule. (**a**) The stage of the 12-cell pro-embryo; (**b**) the stage of the early embryo; (**c**) the stage of the globular embryo; (**d**) the stage of the torpedo-shaped embryo; (**e**,**f**) the stage of the mature embryo. Abbreviations: dz—cell death zone, cu—cuticle, e—endosperm, ent—endothelium, lz—lysis zone, n—nucleus, pa—parenchyma.

Figure 3 shows the ultrastructure details of endothelial cells in the corresponding stages of tomato ovule development. The cells of this newly formed tissue divide, elongate, and function in parallel with the development of the embryo and the surrounding endosperm. Therefore, in all stages, they have an elongated shape and almost the same size. However, significant structural and, accordingly, functional rearrangements take place in them in the process of development.

In the stage of the pro-embryo and eight-cell embryo, the endothelium is still quite tightly adjacent to the endosperm that has begun to develop. Its cells are elongated perpendicular to the surface of the endosperm. The cells exhibit a meristematic structure as observed by transmission electronic microscopy (Figure 3a). In the early embryo stage, the endothelium is separated from the endosperm and a lysis zone is formed between the two tissues (Figure 3b). In the stage of a globular embryo, this lysis zone expands somewhat (Figure 3c). In these stages of development, in the cells of both the endothelium and the endosperm, structures characteristic of secretory cells are formed. One can observe an extensive rough endoplasmic reticulum, sometimes in the form of ergastoplasm, in addition to a hyperactive Golgi apparatus, which is manifested in a large number of dictyosomes. As our previous study showed, both of these tissues, belonging to the mother and daughter organism, are involved in the secretion of mucilage.

In the stage of the torpedo-shaped embryo, a lot of reserve substances have already been deposited in the endosperm, and the membranes of its cells from the side of the endothelium are covered with a cuticle (Figure 3d). Endothelium cells again acquire a structure close to the structure of differentiated cells, but capable of cell division. They retain this structure and properties, until the seed is fully matured.

By the stage of an almost mature embryo, the thickness of the cuticle of endosperm cells increases significantly (Figure 3e). Endothelium cells adhere tightly to the compacted remnants of dead cells of the integument parenchyma, and they are easily separated from the endosperm during dissection (Figure 3f). Thus, it again practically performs the role of the inner epidermis of integument.

### 2.3. Dynamics of the Changes in the Outer Epidermis Cells of Integument

**Figure 4:**
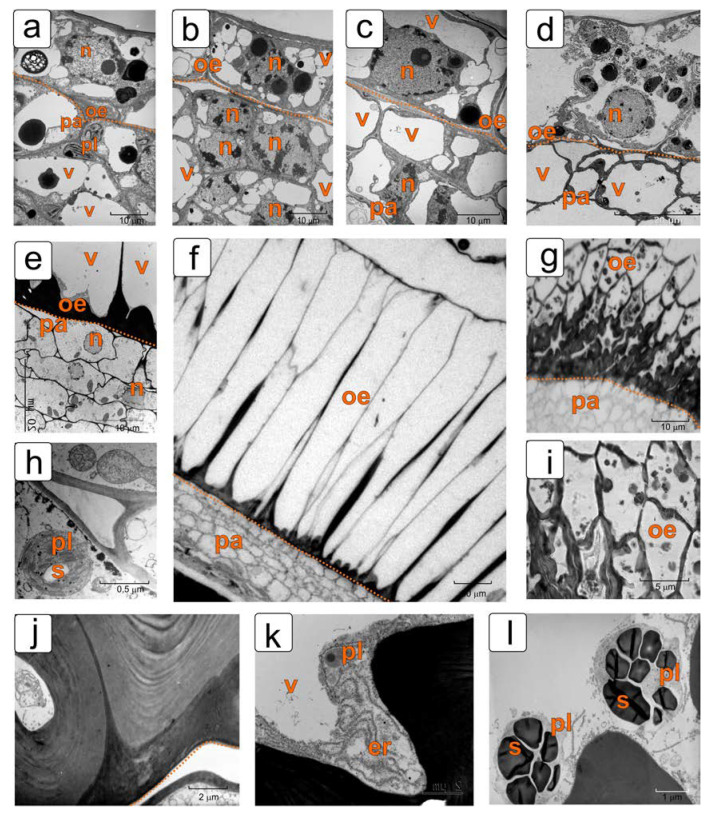
Ultrastructural features of the dynamics of transformations in the outer epidermis of the tomato ovule integument in different stages of ontogenesis. Epidermal cells in the early stages of ovule development: (**a**) the zygote stage; (**b**) the stage of early embryo; (**c**) the stage of globular embryo; (**d**) the stage of torpedo-shaped embryo; (**f**,**g**,**i**) the different stages of mature embryo; (**e**,**h**,**j**,**k**,**l**) ultrastructure fragments of the cells. Abbreviations: oe—outer epidermis, ent—endothelium, er—endoplasmic reticulum, n—nucleus, pa—parenchyma, pl—plastid, s—starch granule, v—vacuole.

Figure 4 shows the structural changes in the cells of the outer integument and adjacent cells of the subepidermal parenchyma.

Until the stage of the torpedo-shaped embryo, the structure of epidermal cells changes insignificantly (Figure 4a–c). They are characterized by a large nucleus in the center and rather large vacuoles in the surrounding cytoplasm. The main feature of epidermal cells (up to the stage of a globular embryo) is the presence of volumetric rounded protein bodies (globoids) in plastids and in vacuoles (Figure 4a–c). In the stage of the torpedo-shaped embryo, the cells of the outer epidermis noticeably enlarge in size. Changes also occur in the internal structure of these cells. Protein bodies become smaller and are observed mainly in the plastids (Figure 4d).

With further growth, the ovules of the outer epidermis cells of the integument begin to elongate perpendicular to the surface and undergo a series of transverse divisions (Figure 4f). The sidewalls become pleated and thicken due to lignification (Figure 4g,i). In the initial stage of this process (Figure 4f), the integument contains 4–5 rows of parenchymal cells with sparse cytoplasm, with nuclei without chromatin and with rare organelles, and elongated epidermal cells, with thickened radial walls at the base (Figure 4e,f). Deposits of the secondary cell wall have a multilayer structure characteristic of most mechanical tissues of plants. Cytoplasm of these cells contains well-developed smooth endoplasmic reticulum (SER), mitochondria, and plastids with osmiophilic granules and small starch grains (Figure 4h,k). In the stage of the almost mature embryo, the envelopes of elongated epidermal cells are strongly thickened, especially at the base (Figure 4j). Different single organelles are observed in the cytoplasm of these cells. Plastids are filled with starch grains (Figure 4l).

**Figure 5:**
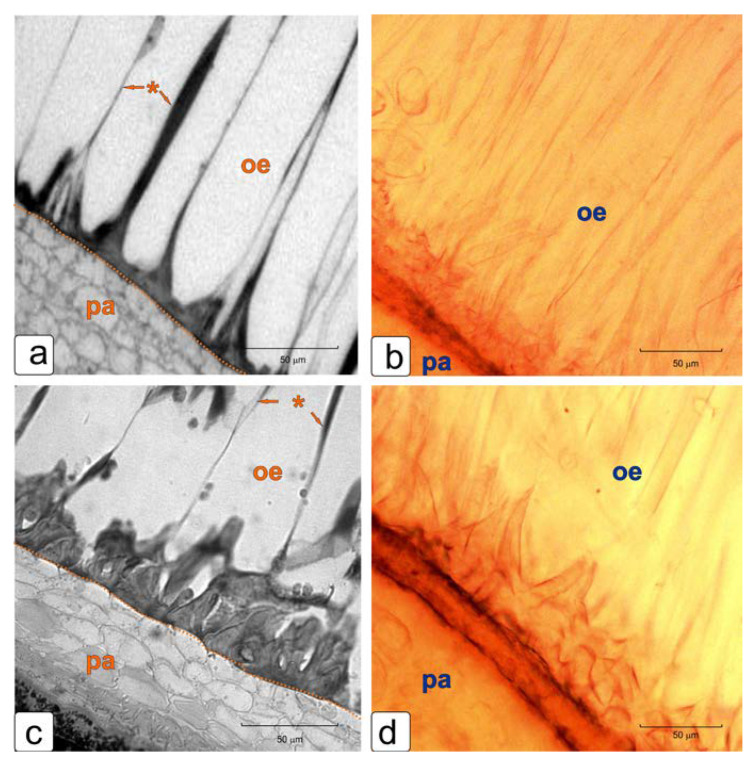
Determination of the presence of lignin in the thickening cell walls of the outer epidermis of integument. Abbreviations: oe—outer epidermis, pa—parenchyma, *—unevenness in the thickness of the cell walls of the outer epidermis. The structure of the cell wall of the crown formed by the outer epidermis in the stage of green fruit (**a**,**b**) and red fruit (**c**,**d**).

The presence of lignin in the thickening of the elongated cell walls of the integument epidermis in the late stages of seed formation was confirmed by staining with phloroglucinol (Figure 5). We can observe a gradual increase in lignification.

Elongated giant cells of the outer epidermis form a complex cell wall, which have a thickening in the lower part due to the formation of a secondary cell wall, most pronounced at the border with the parenchyma (Figure 4f–l; Figure 5a,c). The cell wall along its entire length forms extended sections with thickenings, which later intensively lignify (Figure 5b,d). Probably, this structure ensures the ease of formation of seed pubescence, which is typical for tomato (Figure 1g,h), but with careful extraction of seeds, it has the character of partial tears (Supplementary Materials Figure S1).

### 2.4. Ricinosomes as a Probable Prognostic Trait of Integument Parenchyma PCD

From the outset of the cell death in the integument, ricinosomes are found in its dying cells. Therefore, the presence of ricinosomes has been recognized as an early trait that can be used to predict PCD during tissue development.

**Figure 6:**
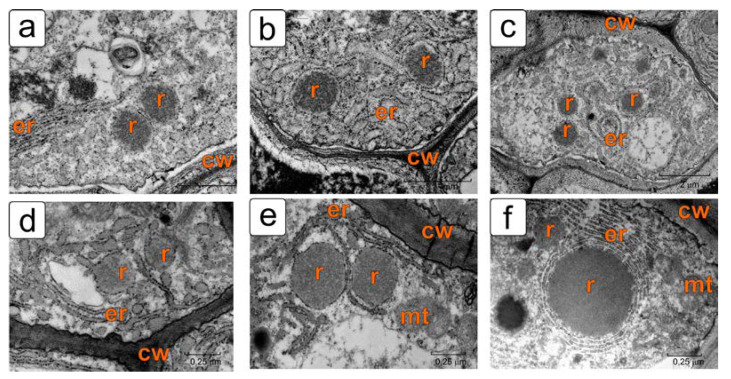
Integument cells with ricinosomes from the cell death zone in the initial stages of tomato ovule development: (**a**) the stage of the pro-embryo; (**b**,**c**) the stage of the early embryo; (**d**,**e**,**f**) the stage of the globular embryo. Abbreviations: cw—cell wall, er—endoplasmic reticulum, mt—mitochondrion, pl—plastid, r—ricinosome.

The appearance of ricinosomes in the parenchyma cells of integument, near the inner epidermis, correlates with the onset of PCD of these integument cells in the tomato ovule. The disintegrating cells separate the integument from the epidermis and it converts into an independent monolayer tissue—the endothelium (Figure 6a).

Ricinosomes are detected in the cells of inner layers of the tomato ovule integument immediately after fertilization (Figure 6a). In the early stages of tomato ovule development, ricinosomes in the integument cells in the cell death zone have a typical appearance for these organelles: an electron-dense central part and an electron-transparent region adjacent directly to the surrounding membrane with ribosomes attached to it (Figure 6a–c).

In these cells, other characteristic features of PCD are also observed. The sinuous outlines of the plasma membrane indicate the beginning of protoplast compression. However, the cytoplasm has a rather dense structure; it is filled with a large number of cisternae of the rough endoplasmic reticulum (ER). In some cells, along with a narrow ER profile, short dilated cisternae are present (Figure 6a). The cytoplasm of other cells is filled with a network of dilated ER cisternae (Figure 6b). Cells with typical narrow ER profiles are also often seen (Figure 6c). Homogeneous ricinosomes, without a peripheral layer, are observed in those cells where ricinosomes are probably still in the stage of formation and are connected to the cisternae of the reticulum. Mitochondria in such cells appear swollen (Figure 6d–f). Some cells also contain ricinosomes surrounded by ER rings in the form of ergastoplasm (Figure 6f).

**Figure 7:**
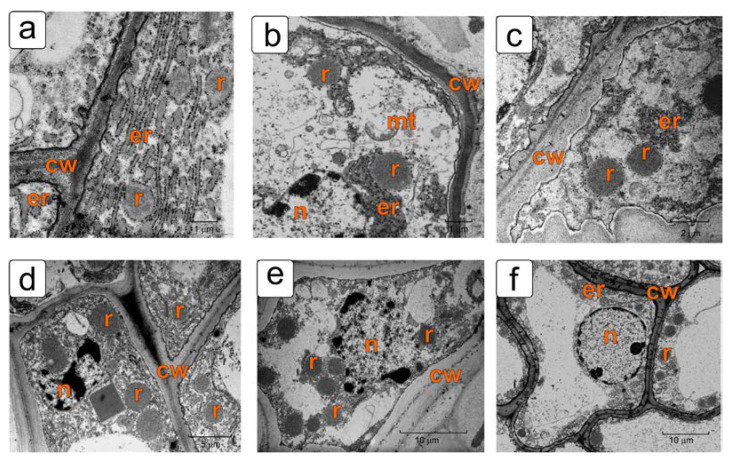
Integument cells with ricinosomes from the cell death zone in later stages of tomato ovule development: (**a**–**d**) the stage of globular embryo; (**e**,**f**) the stage of torpedo-shaped embryo. Abbreviations: cw—cell wall, er—endoplasmic reticulum, mt—mitochondrion, pl—plastid, r—ricinosome.

In later stages, the integument cells with ricinosomes have a more transparent cytoplasm and a small number of ricinosomes of typical shape and size. Ricinosomes in these cells are associated with narrow ER cisternae, which are unevenly distributed in the cell (Figure 7a–c). In most cells, the plasmatic membrane is observed to separate from the cell walls to one degree or another, accompanied by compaction of the outer cell envelope (Figure 7b,c).

Along with ricinosomes, protein bodies in the form of crystalloids of various sizes and shapes—square, triangular, and trapezoidal—are often observed in degrading integument cells from the cell death zone. They are located both in the cytoplasm and in the vacuoles (Figure 7d, Supplementary Materials Figure S2).

### 2.5. Modifications of Dying Integument Cells at the Stages of Early and Globular Embryo

**Figure 8:**
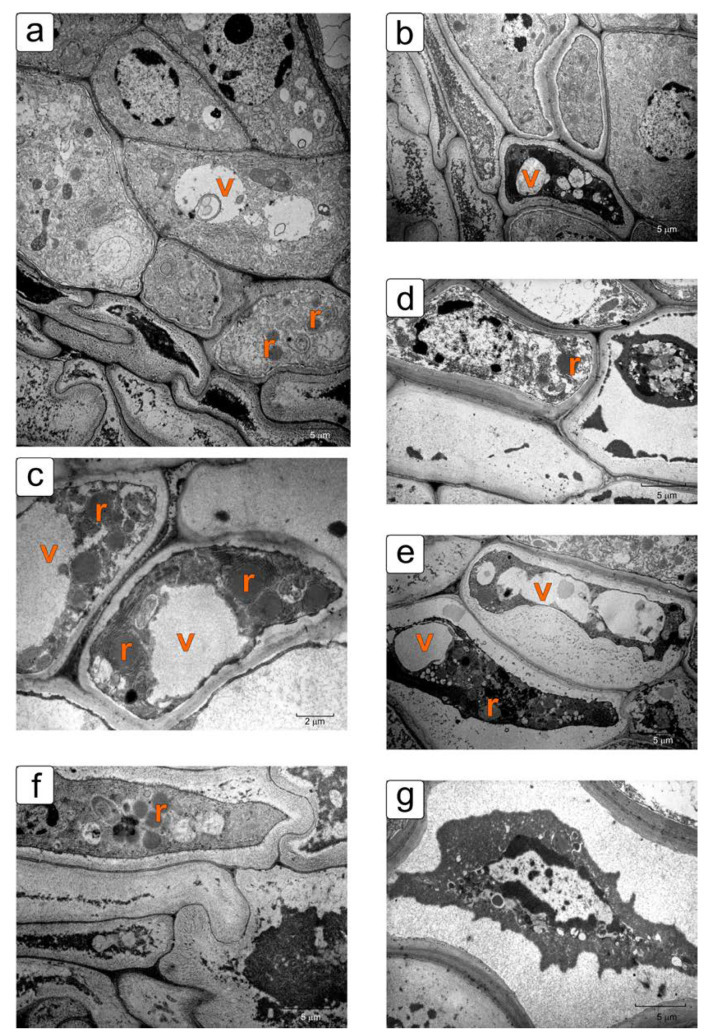
Fragments of cells of the integument parenchyma in different stages of the cell death (stages of early and globular embryo). The photographs show almost all stages of death: vacuolization, discharge of the plasmatic membrane, compression of the protoplast, collapse of the protoplast, and then lysis: (**a**,**b**) general picture of almost all stages of death; (**c**–**g**) fragments of the final stages of this process. Abbreviations: v—vacuole; r—ricinosome.

Figure 8 shows microphotos of different stages of integument cell death in the stages of the early and globular embryo.

An increase in the number of signs of PCD is traced to the integument parenchyma cells from the outer row of cells to the inner one, namely: increased vacuolization, an increase in the number of ricinosomes, and the detachment beginning of the plasmatic membrane from the cell wall (Figure 8a). Below these cells there is a layer of cells with compressed dead protoplasts, and, even lower, only cell envelopes with almost completely lysed protoplasts. Figure 8b shows the process of protoplast compression and degradation, and Figure 8d shows the lysis. Other photos show different variants of these processes. It should be noted that ricinosomes in degrading cells do not burst, do not change their appearance, and, together with other organelles of the protoplast, are compressed and then lysed (Figure 8c–f).

Along with ricinosomes, protein bodies in the form of crystalloids of various sizes and shapes—square, triangular, and trapezoidal—are often observed in degrading integument cells from the cell death zone. They are located both in the cytoplasm and in the vacuoles (Figure 7d, Supplementary Materials Figure S1).

### 2.6. Protoplasts Destruction Process of Integument Parenchyma Cells, Adjacent to the Inner Epidermis

**Figure 9:**
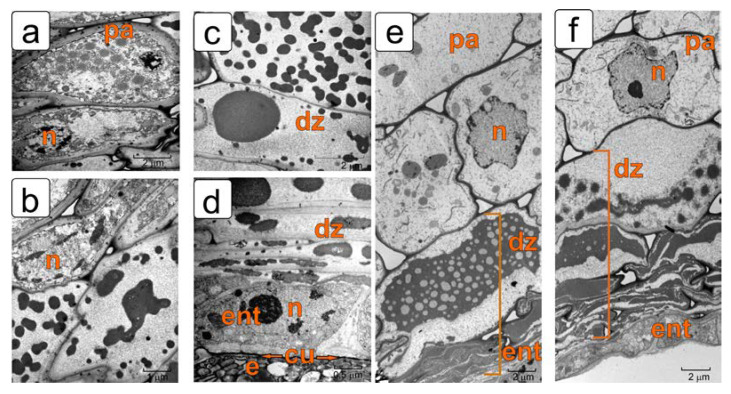
The process of death of integument cells in the stage of the torpedo-shaped embryo and in the stage of mature tomato seed. Stages of death: (**a**) cell with ricinosomes and nearby cell with the beginning of protoplast compression; (**b**) further compression and cleavage into vesicles; (**c**) association of vesicles into solid bodies; (**d**) flattening of cells and compression near the endothelium; a cuticle layer is formed on the surface of the endosperm; (**e**,**f**) final stages of death and contraction of integument cells. Abbreviations: e—endosperm, ent—endothelium, n—nucleus, pl—plastid, r—ricinosome, vs—vesicles.

In the final stages, the presence of a large number of ricinosomes indicates the beginning of the PCD process in the parenchyma cells of integument (from the side of the inner epidermis, which acts as an endothelium with high secretory activity) (Figure 9a). Then the compression of half-destroyed protoplast occurs into a total mass, wherein, the material shifts to the center of the cells, which is accompanied by an increase in the thickness of the internal volume of the cell walls. Further, the biomass of the dense contents of the cell is split into separate vesicles having a round shape, which indicates that their density becomes higher than the surrounding material. Then, these vesicles unite again, forming one or more dense bodies inside the flattened cell walls, since lysis does not occur. Such processes may indicate a change in the osmotic adjustment accompanying this process. Layers of dead cells are directly adjacent to the endothelium (Figure 9d). The endothelial cells themselves no longer show signs of secretory tissue and hardly change. They have mainly the structure of differentiated functioning cells, with a high level of metabolism.

In the process of seed maturation, the layers of flattened cells are further compressed and closely adjoin the endothelium (Figure 9e,f). Thus, we can assume that the endothelium returns to its location and again becomes the inner epidermis of integument with the corresponding structure.

Thus, by the time of the full development of the tomato seed, only the outer epidermis, which is highly modified with thickened lignified membranes, and 2–3 layers of subepidermal cells, remain from the integument on the outside. On the inside, they are adjoined by layers of the remnants of dead cells of the integument parenchyma flattened into a total mass and the outer epidermis (endothelium), which has returned to its functions and to its location. Together, these layers form a complex multi-layered skin of a mature tomato seed. Thus, it was possible to fully trace the dynamics of transformation of the single integument into a protective coat of tomato seeds.

## 3. Discussion

The main goal of this study was to study the morphological and ultrastructural details of the formation of a sufficiently strong tomato seed coat (skin) and to identify which ovule tissues are involved in this process. Since the ovule development is inevitably associated with the death of the integument parenchyma cells, the description of the ultrastructure details of this process is part of this work.

The seed coat (outer skin of the seed) of most flowering plants is formed from the maternal tissues surrounding the embryo sac. These tissues undergo various changes during ontogenesis, forming a multilayered coat of a mature seed [3,5].

Seed development occurs as a result of the differentiation of new tissues, and the orderly disappearance of some maternal tissues [6,22,23], which contributes to the free growth of a new sporophyte. However, the different tissues undergo PCD in ovules of different types: one-integumentary (unitegmic) and two-integumentary (bitegmic). In bitegmic ovules, free space for embryo growth is created by PCD of nucellus cells [12,24,25,26]. As a rule, both integuments are involved in the formation of the seed coat in such ovules. In some cases, one integument is eliminated. For example, in cereals, the outer integument disappears during the ovule development and, in addition to the two layers of the inner integument, three more layers of pericarp cells are involved in the formation of caryopsis (kernel) skin [27,28,29,30].

In most unitegmic ovules, the nucellus degrades at the earliest stages and is utilized by the developing embryo sac. Therefore, the expansion of space for the growing embryo and the endosperm in such ovules occurs due to the PCD of the inner layers of a single integument. The most noticeable and characteristic signs of PCD in plant cells are DNA degradation, in addition to separation of the protoplast from the cell wall and protoplast contraction. In many cases, this process is preceded by the appearance of ricinosomes [31].

However, cell death in different structures of the developing seed may occur in different ways, which is probably due to the peculiarities of the processes of cell differentiation. Therefore, one type of PCD can be fundamentally different from other types of PCD in plant tissues [15]. Several types of PCD are believed to exist in plant cells, but many of them are still unknown [32]. Even within the same type of ovule, the death of similar tissues can occur in completely different ways. For example, the zone of death of integument cells in unitegmic ovules of most representatives of the *Compositae* family is formed due to mucilage of the cells in the inner layers of the integument parenchyma. As a result, the integument cells that are destroyed in this way form a mucoid space for the unhindered growth of embryo [33,34,35,36,37].

In the unitegmic tomato ovule, already at the very initial stages of development, in the cells of the inner layers of the integument, morphological markers of PCD, ricinosomes, formed from enlargements of the ER, are found. These organelles are found during PCD in aging cells of different ovule tissues under plant ontogeny [12,24,25,38]. It has been observed that ricinosomes are usually found in cells before the appearance of other changes characteristic of PCD [39,40,41]. It was also concluded that the presence of ricinosomes in cells can be used to predict PCD [31]. Ricinosomes are spherical organelles derived from the endoplasmic reticulum. They contain pro-cysteine endopeptidases (CysEP) that have a wide range of targets. These targets are various proteins, which are degraded during PCD [42].

Currently, ricinosomes have been found only in plants [31,43]. It was the presence of ricinosomes in integument cells adjacent to the inner epidermis that allowed us in our previous study to determine how the endothelium separates from the integument in the tomato ovule, since this was not previously understood [11].

According to the present study, ricinosomes are present in fairly large numbers in the cells of the tomato ovule integument at all stages of development. As a rule, they have a typical structure, shape, and size. In this form, ricinosomes are observed even inside compressing cell protoplasts in the zone of death. We did not observe fusion of ricinosomes or their disintegration, as was shown in some other tissues [12]. Thus, the main observation is that the ultrastructure of ricinosomes almost does not change at different stages of the tomato ovule development, wherein, PCD traits progress in cells with ricinosomes: a decrease in the electron density of cytoplasm; wrinkling of the plasma membrane; detachment of plasma membrane from the cell wall; and, finally, the compression of the protoplast.

In the present work, along with the identification of PCD features of the parenchyma cells of integument, we sought to study the details of transformation of the outer and inner epidermis cells during the formation of the tomato seed coat. The analysis of all the results obtained made it possible to present a complete pattern of the relationship between all parts of the single integument of tomato ovule. This made it possible to reveal that PCD of the parenchyma cells of integument is an important part of the process of the tomato seed coat formation.

As already noted, the integument differentiation in the tomato ovule ontogenesis is determined by two synchronously occurring processes—an increase in the mass of cells from the outside and their destruction from the side of the embryo sac. Therefore, it was important to reveal the degree of coherence of morphological rearrangements between the outer and inner zones of integument during these processes. A comparison of all morphological and, especially, ultrastructure data made it possible to single out two main stages under the formation of the tomato seed coat, and to reveal important details about the transition from one stage to another.

### 3.1. The First Stage—From Fertilization to the Formation of Globular Embryo

Primarily, it should be noted that the tomato ovule is arranged quite simply. Its only integument consists of several layers of thin-walled vacuolated cells, bounded by two epidermises—outer and inner, wherein, the inner epidermis adjoins directly to the embryo sac. In this case, the embryo sac does not have any additional protective layer.

After fertilization, the processes of embryogenesis and endosperm genesis are triggered in the embryo sac of the tomato ovule. Therefore, a space is required for the active growth of a new sporophyte (embryo and endosperm). This space and its expansion are provided by the programmed death of the inner cell layers of integument. However, the inner epidermis is not involved in this process. Due to the death of the layer of parenchyma cells adjacent to it, it is detached from the integument. It seems that the integument itself separates its inner epidermis from itself. After distancing from the integument, the inner epidermis is transformed into a single-layer distinct tissue—the endothelium. This tissue becomes a living protective barrier for the developing new sporophyte. This amazing process is also described in a previous work [11]. It is important to note the fact that at this stage of development, endothelial cells acquire the internal structure of secretory cells. In this regard, we can say that such a transformation of this tissue can serve as an example of evolutionary convergence [44].

Therefore, dying parenchyma cells undergo almost complete lysis, which is necessary for the rapid expansion of space for the growing embryo sac. Simultaneously with these processes, the number of cell layers and, accordingly, the thickness of the integument itself, continue to increase, reaching its maximum size in the stage of globular embryo. In this stage, cells of the outer epidermis mainly proliferate and, as a result, do not undergo any noticeable morphological and structural changes. Thus, the main feature of the first stage of differentiation of the tomato ovule and, accordingly, its coat, is that the total number of cell layers of the integument from the outside increases simultaneously with the expansion in the so-called zone of the lysis of dead cells. The endothelium plays an important role in this process. The former name of this tissue was the integumentary tapetum. Previously, researchers reported on the secretory role of this tissue in the ovules of various plants [45,46].

### 3.2. The Second Stage—From Torpedo-Shaped Embryo to Maturation

This stage is characterized by the beginning of organogenesis in the embryo. The embryo is actively growing, and the endosperm cells are filled with storage substances. A cuticle appears on the surface of the endosperm. This means that the emerging young sporophyte separates from maternal tissues and further growth of the embryo occurs mainly on account of the use of endosperm cells. Therefore, the interaction of the endosperm with the endothelium weakens, and after further thickening of the cuticle, it stops altogether. As a result, the structural organization of endothelium cells again undergoes changes, but now in the direction of simplification. These cells again acquire meristematic features, thus losing their secretory functions. This is what leads to a significant change in the fate of dying cells of the integumentary parenchyma. In addition, first of all, the process of converting of compressed protoplasts is completely changed. They no longer lyse, but first break up into small vesicles, then again clump into a common, denser mass. Perhaps this is a way to more firmly compact dead protoplasts. After all, as a result, such cells form dense layers, from which the inner mechanically strong part of the coat of a mature tomato seed is formed. The endothelium adjacent to this layer, as it were, returns to the location of the inner epidermis and remains unchanged almost until the seed is completely mature. Perhaps it plays the role of a moisturizing layer on the inside of the coat.

The differentiation of the outer epidermis of the integument, characteristic of tomato seed, is activated only at the stage of the torpedo-shaped embryo. First, the cells of the epidermis increase in size (in comparison with the previous stages), and then they gradually stretch in length perpendicular to the surface of the ovule. It has been shown that the side walls at the base of these cells thicken due to lignification. They have the internal structure of living, functioning cells. Only 2–4 layers of cells remain of the integumentary parenchyma in this zone, containing transparent cytoplasm with rare organelles and without vacuoles. Before full maturation of the seed, the walls of these cells are slightly lignified.

Thus, at the second stage, there is a gradual maturation of the embryo and the formation of all layers of the tomato seed coat. A compacted congestion of dead cells of the integument forms a strong inner frame layer of this coat, and the outer epidermis is transformed into a kind of openwork frame of the seed.

The outer epidermis of any plant organ it is known to serve to protect against the adverse environment. Therefore, the structure of epidermis must be adapted to the environment in which development occurs [47,48]. Considering that tomato seeds are formed in a humid environment inside a mellow fruit, we assume that this circumstance was the reason for such an unusual differentiation. However, this issue requires further research.

The unitegmic integument of tomato ovule contributes, despite the absence of the second integument, to the formation of a complex multifunctional coat, which is characteristic of both unitegmic and bitegmic ovules, which is also reported for other species [48,49].

Thus, in this study, for the first time, all the structural features of the formation of the tomato seed coat at different stages of development were studied and analyzed in detail, and the degree of participation of each tissue in this process was shown. A generalized analysis of the data showed that during the tomato seed coat formation, there is a clear correlation in the differentiation of the outer and inner sides of tomato ovule integument.

## 4. Materials and Methods

### 4.1. Plant Material

Plants of tomato (*Solanum lycopersicum* L.) cv Belyi Naliv were grown in a climatic chamber under the following environmental conditions: light/dark cycle of 16/8 h, temperature of 24/20 °C, light intensity of 200 μmol m^−2^s^−1^, and 60% relative humidity. To improve fertilization, the flowers were shaken. After fertilization, flowers and developing fruits were taken in different periods of ovule development from 0 (the first day after fertilization) to 50 days (full maturation of the fruit).

### 4.2. Sample Fixation and Preparation

Isolated ovules in different stages of development were fixed for 24 h in 2.5% glutaraldehyde (Merck, Darmstadt, Germany) prepared in 0.1 M Sorensen phosphate buffer (pH 7.2) and containing 1.5% sucrose. Samples were thoroughly washed with buffer, then additionally fixed in 1% OsO4 (Sigma-Aldrich, St. Louis, MO, USA) and dehydrated in increasing concentrations of ethanol (30, 50, 70, 96 and 100% for 30 min each). To improve the quality of the sample preparation, propylene oxide was used and poured into a mixture of Epon-812 and Araldite (Merck, Darmstadt, Germany) according to the standard protocol. Then, the samples were placed in filling capsules with the addition of a catalyst and subjected to two-stage polymerization. For light microscopy, semi-thin sections were prepared using an ultramicrotome LKB-V (LKB, Bromma, Sweden). Sections (1–2 μm) were placed on glass slides and stained with 0.1% methylene blue (Merck, Darmstadt, Germany). After that, permanent preparations were prepared—they were placed in epoxy resin under cover glasses. Samples were analyzed and photographed using an Olympus BX51 microscope (Olympus, Shinjuku, Tokyo, Japan) equipped with a Color View II camera (Soft Imaging System, Münster, Germany). At least 300 samples of mentioned above ovule tissues from three independent plants were analyzed.

### 4.3. Method for the Determination of Lignin

To determine the localization of lignin in the tissues of the shell of tomato seed, a 1% solution of phloroglucinol (Biochem, Cosne Cours sur Loire, France) in 50% ethanol and concentrated hydrochloric acid in a ratio of 3:1 were used. The isolated fragments of the outer epidermis of the seed of fully formed fruits of green and red tomato fruit were used. They were placed on a glass slide in a phloroglucinol solution for 10 min, then, the liquid was aspirated with filter paper, after which a drop of HCl was added and covered with a cover glass. After that, the solution was replaced with glycerin. A characteristic red color developed due to the presence of coniferaldehyde groups in lignin. The preparations were viewed under ×10 magnification on an Olympus BX51 microscope (Shinjuku, Tokyo, Japan) with the Cell program.

### 4.4. TEM Analysis

For electron microscopy, samples were made using a diamond knife with a width of 1.5 mm on an LKB-V ultramicrotome (LKB, Bromma, Sweden). Sections were placed on formvar coated blends or grids and stained with uranyl acetate and lead citrate. Then the thin sections were analyzed and photographed using H-500 (Hitachi, Ibaraki, Japan) and JEM-1400 (Jeol, Akishima, Tokyo, Japan) electron microscopes.

### 4.5. Sample Preparation for Scanning Microscopy Analysis

The dry tomato seeds were placed onto a SEM stub, which is covered by carbon adhesive tape (Double-Sided Carbon Tape, 8 × 20 mm, EMS Cat#77817-08-AL, Shanghai, China) and coated with gold and palladium using an Eiko IB-3 ion-coater (Eiko, Tokyo, Japan). The thickness of the coated layer was 20 nm. Then the seeds were observed under a Camscan-S2 SEM (Cambridge Instruments, Cambridge, UK) in the Laboratory of Electron Microscopy (Biological Faculty of Lomonosov Moscow State University).

## 5. Conclusions

New data were obtained on the details and dynamics of transformation of the integument of a unitegmic (one-integumentary) ovule of tomato into the mature seed skin. It was shown that both epidermis of the integument, both outer and inner, play an important role in this process. Their differentiation occurs synchronously with the differentiation and formation of the embryo and endosperm. However, in accordance with the opposite location of each epidermis, their differentiation and functional orientation are fundamentally different. The research demonstrated in detail that the inner epidermis after fertilization separated from the integument through PCD of neighboring cells of the integument parenchyma and is transformed into a new independent monolayer tissue—endothelium. This tissue is necessary to protect the embryo and endosperm from the destructing cells zone of the integument parenchyma.

Throughout almost the entire further development of the ovule, the endothelium grows next to the growing endosperm. During this time, its cells practically do not change their shape and size, but change the ultrastructure several times, and, accordingly, the functional orientation changes from meristematic (after separation from the integument parenchyma) to secretory (in the stage of the globular embryo), and then again to meristematic and, finally, to differentiated. These observations explain the fact that dying integument cells are initially almost completely lysed, and later, in the absence of lysis, a simple accumulation of flattened dead integument cells occurs. In this final period of tomato seed development, the endothelium returns to its original location in the inner epidermis of the already strongly altered integument. The death of cells of the integument parenchyma is preceded by the appearance of specialized organelles in them—ricinosomes, which are markers of PCD.

## Data Availability

Not applicable.

## Supplementary Materials

The following supporting information can be downloaded at https://www.mdpi.com/article/10.3390/plants11091101/s1, Figure S1: Scanning electron microscopy of a tomato seed surface; Figure S2. Integument cells with protein crystalloids.
