# Supplementary material for: Morphological and Structural Details of Tomato Seed Coat Formation: A Different Functional Role of the Inner and Outer Epidermises in Unitegmic Ovule
Source: Plants (Basel). 2022 Apr 19;11(9):1101. doi: 10.3390/plants11091101 (PMC9104524; doi:10.3390/plants11091101)
Supplement: Supplementary file 1 [file plants-11-01101-s001.zip › plants-1676140-supplementary.pdf]

Supplementary Materials.

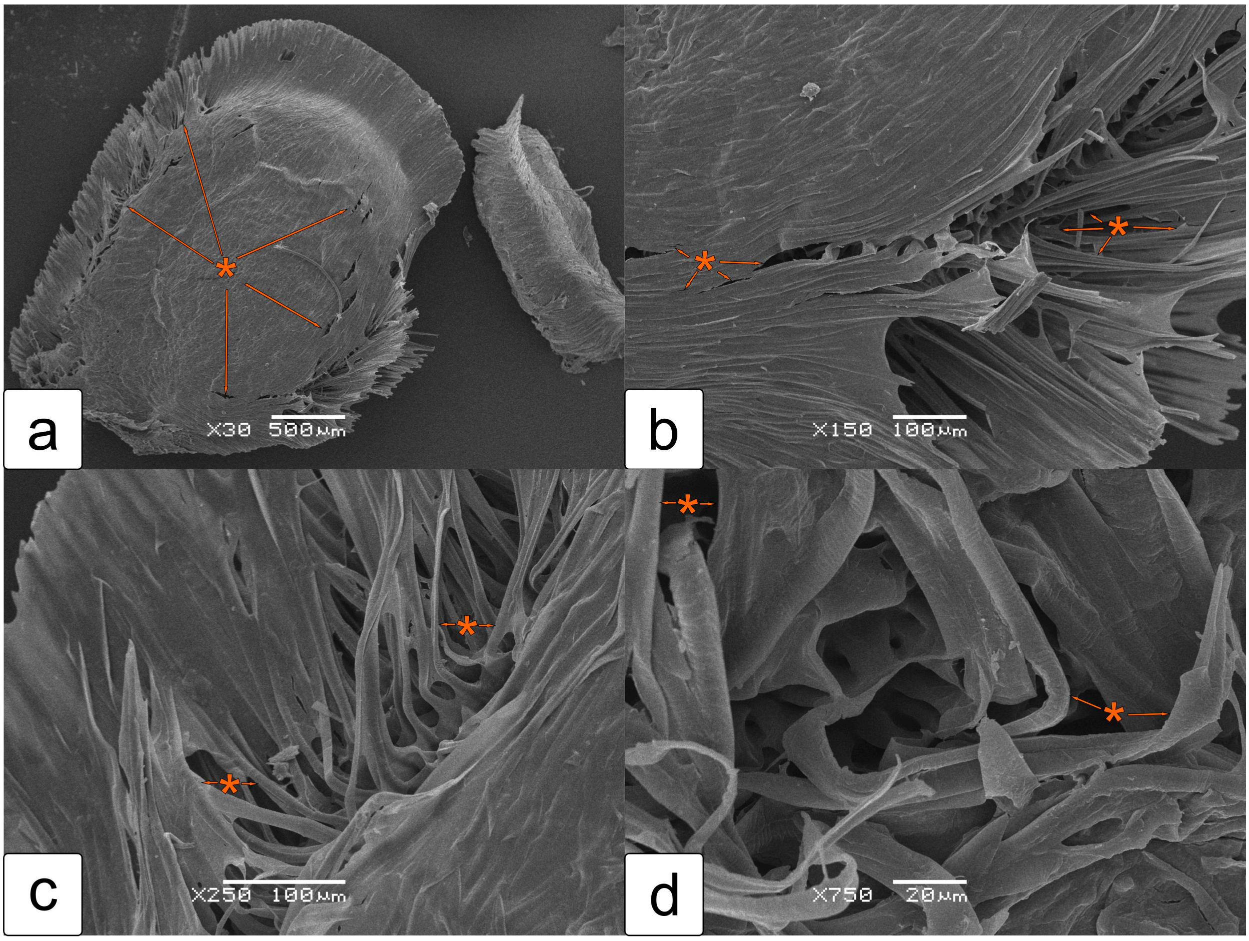

Figure S1. Scanning electron microscopy of a tomato seed surface. Forming cell ruptures of the outer epidermis caused a fleecy surface: * - longitudinal separation of the cell walls in giant epidermal cells after seed drying.

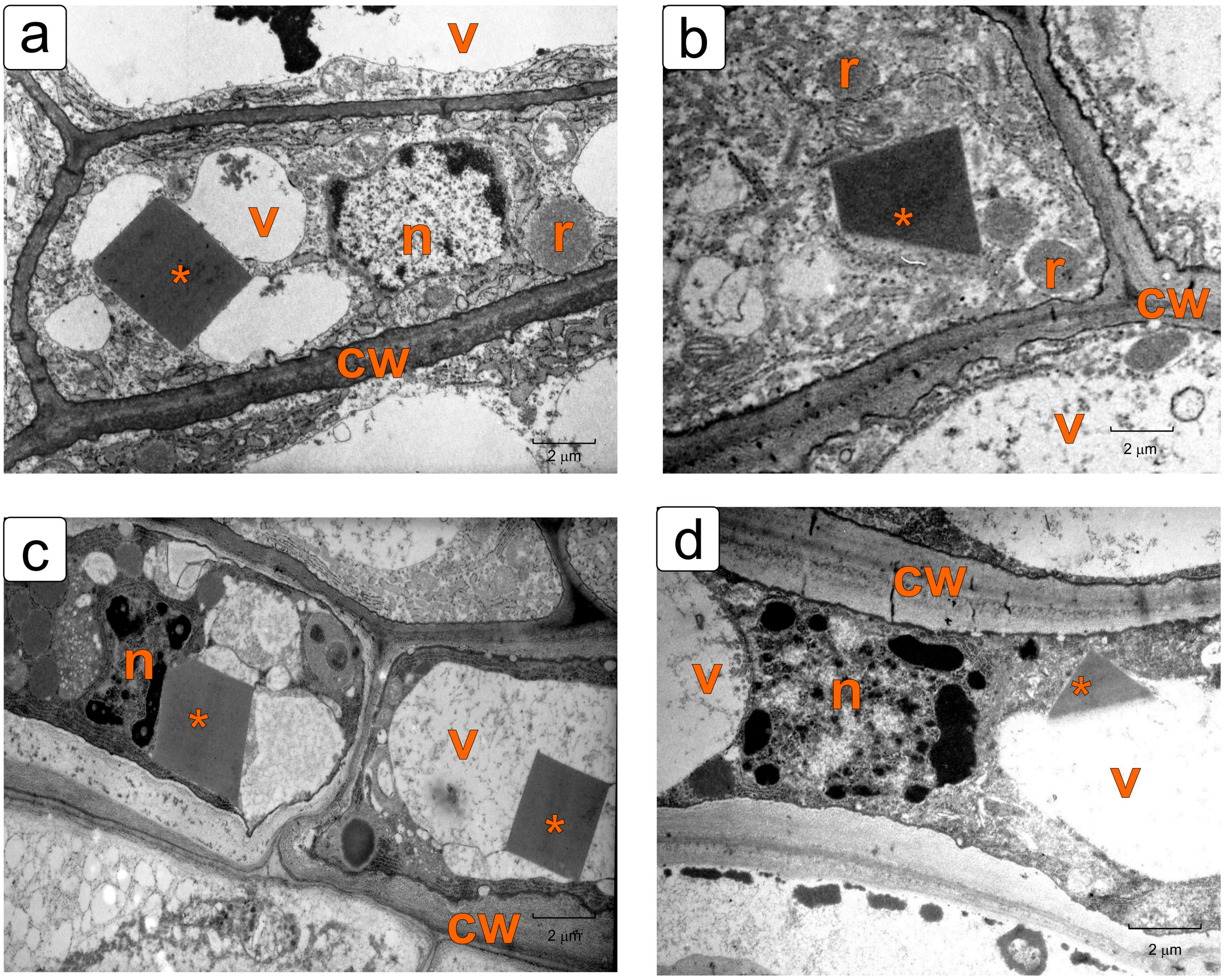

Figure S2. Integument cells with protein crystalloids. Crystalloids are observed in dying integument cells in different stages of seed development.
